# Scrap and Build for Functional Neural Circuits: Spatiotemporal Regulation of Dendrite Degeneration and Regeneration in Neural Development and Disease

**DOI:** 10.3389/fncel.2020.613320

**Published:** 2021-01-11

**Authors:** Kotaro Furusawa, Kazuo Emoto

**Affiliations:** ^1^Department of Biological Sciences, Graduate School of Science, The University of Tokyo, Tokyo, Japan; ^2^International Research Center for Neurointelligence (WPI-IRCN), The University of Tokyo, Tokyo, Japan

**Keywords:** dendrite, remodeling and dysfunction, morphogenesis, development, repair

## Abstract

Dendrites are cellular structures essential for the integration of neuronal information. These elegant but complex structures are highly patterned across the nervous system but vary tremendously in their size and fine architecture, each designed to best serve specific computations within their networks. Recent *in vivo* imaging studies reveal that the development of mature dendrite arbors in many cases involves extensive remodeling achieved through a precisely orchestrated interplay of growth, degeneration, and regeneration of dendritic branches. Both degeneration and regeneration of dendritic branches involve precise spatiotemporal regulation for the proper wiring of functional networks. In particular, dendrite degeneration must be targeted in a compartmentalized manner to avoid neuronal death. Dysregulation of these developmental processes, in particular dendrite degeneration, is associated with certain types of pathology, injury, and aging. In this article, we review recent progress in our understanding of dendrite degeneration and regeneration, focusing on molecular and cellular mechanisms underlying spatiotemporal control of dendrite remodeling in neural development. We further discuss how developmental dendrite degeneration and regeneration are molecularly and functionally related to dendrite remodeling in pathology, disease, and aging.

## Introduction

Dendrites are specialized structures designed to receive information from presynaptic neurons or sensory organs. During postnatal development of the mammalian brain, neurons exhibit extensive plasticity in which connectivity can be modified in response to neural inputs and/or hormonal regulation (Parrish et al., 2007a; Jan and Jan, 2010; Emoto, 2011; Batista and Hensch, 2019; Molnar et al., 2020). To achieve these changes in connectivity, neurons often remodel their dendrite shape through a combination of degeneration and regeneration of local dendritic branches (Kanamori et al., 2015b; Riccomagno and Kolodkin, 2015). Owing to technical advances in *in vivo* imaging, researchers are now able to fully trace branch dynamics of single neurons with high spatiotemporal resolution. These *in vivo* imaging studies have revealed that developing dendrites often undergo multiple rounds of regeneration and regeneration before the establishment of their final shape (Yasunaga et al., 2010; Kaneko et al., 2011; Takeo et al., 2015; Nakazawa et al., 2018).

In addition to developmental dendrite degeneration and regeneration, certain types of neurons remodel their dendritic arbors in response to injury on dendritic branches (Richardson and Shen, 2019; Liu and Jan, 2020). The progression of injury-induced dendrite degeneration and regeneration are morphologically similar to what was observed in developmental dendrite degeneration and regeneration, respectively, suggesting that the developmental and injury-induced remodeling involve a shared program. However, recent studies indicate that regulatory mechanisms of injury-induced dendrite degeneration and regeneration are distinct at least in part from mechanisms governing either developmental dendrite degeneration and regeneration or injury-induced axon degeneration and regeneration (Stone et al., 2014; Thompson-Peer et al., 2016; Hao et al., 2019; Zhu et al., 2019).

In this review article, we first describe an overview of diverse types of dendrite degeneration and regeneration in vertebrates and invertebrates. We then review what is currently known about the molecular and cellular mechanisms underlying dendrite remodeling, focusing on the temporal and spatial control of degeneration and regeneration. We also discuss how developmental dendrite degeneration and regeneration are molecularly and functionally related to dendrite remodeling in pathology, disease, and aging.

## Developmental Dendrite Remodeling in Vertebrate Neurons

**Figure 1:**
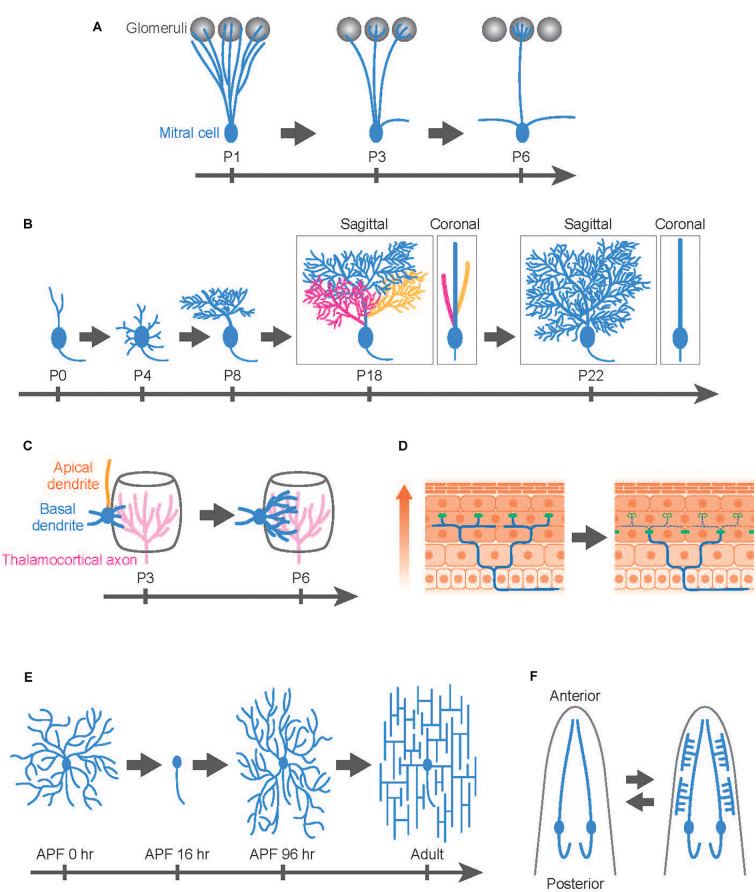
Developmental dendrite remodeling in a variety of neurons. Dendrite remodeling in mitral cells in the murine olfactory system **(A)**, Purkinje cells in the murine cerebellum **(B)**, layer IV pyramidal neurons in the murine somatosensory cortex **(C)**, dorsal root ganglion (DRG) neuron innervating the murine skin **(D)**, *Drosophila* class IV dendrite arborization (C4da) sensory neurons **(E)**, and *C. elegans* IL2 sensory neurons **(F)**. **(A)** Mitral cells initially innervate dendrites (blue) to multiple glomeruli (gray) and later eliminate all but one apical dendrite. **(B)** Layer IV pyramidal neurons (spiny stellate neurons) initially extend both apical (orange) and basal (blue) dendrites followed by retraction of the apical dendrites while further extending basal dendrites toward thalamocortical axons (magenta). **(C)** Purkinje cells develop multiple dendritic protrusions from the soma, followed by elimination of the whole branches in the first postnatal week. In the subsequent postnatal development, Purkinje cells elaborate multiplanar dendrites in a 3D space (P18: three colors represent different dendritic branches arising from the soma), then eventually confine the trees into a 2D space (blue) by trimming branches (P22: Branches with yellow and magenta colors have been eliminated during this period). Both sagittal and coronal views are shown for P18 and P22 images. **(D)** Mammalian skin is composed of multiple layers of epidermal cells. Epidermal cells are continuously generated from stem cells and move toward the upper layer of the skin (arrow). According to this epidermal cell turnover, the tight junctions (green dots) are also remodeled in the deep epidermal layer. DRG neurons typically maintain their sensory terminals (blue) underneath the tight junctions by extension, retraction, and pruning of the nerve ends. **(E)** *Drosophila* C4da neurons replace their larval dendrites with adult-specific arbors during metamorphosis. After establishing their dendritic fields in the pupal development, C4da neurons immediately reshape the dendritic arbors from the radial to the lattice-like structures in the first 1–2 adult days. APF; after pupal formation. **(F)** *C. elegans* IL2 sensory neurons typically elaborate simple unbranched dendrites in the normal condition. In response to adverse environmental conditions, however, IL2 neurons undergo dendrite remodeling, shifting from a bipolar to a multipolar state. This process is reversible: the arborized dendrites are pruned away after worms are returned to the normal (non-stressful) environment (bidirectional arrows). Anterior is shown at the top.

Developmental dendrite remodeling is typically achieved by degeneration and regeneration of local dendrite branches. One well-studied system is the dendrite remodeling of mitral cells, the second-order projection neurons in the mammalian olfactory system (Wong and Ghosh, 2002). In the adult olfactory bulb, mitral cells extend a single apical dendrite radially that arborizes a tuft within one glomerulus (Mori and Sakano, 2011; Sakano, 2020). Also, mitral cells extend lateral dendrites that are widely distributed within a horizontal plane in the external plexiform layer and make reciprocal dendrodendritic synapses with granule cells. This mature arborization pattern is the result of extensive refinement: during perinatal development, mitral cells extend dendritic branches to multiple glomeruli, and subsequently, they lose all but one dendritic branch, maintaining contacts with a single glomerulus as they mature (Figure 1A; Mori and Sakano, 2011; Murthy, 2011; Sakano, 2020). This selective dendrite degeneration in mitral cells is critical to fine-tuning olfactory circuits involved in odor processing (Inoue et al., 2018; Fujimoto et al., 2019).

Purkinje cells in the cerebellum typically elaborate space-filling type dendrites in a single parasagittal plane (Figure 1B), but as in the case of mitral cells, the mature form of Purkinje cell dendrites involves developmentally programmed degeneration and regeneration. Recent *in vivo* imaging studies have revealed that Purkinje cells establish distinct features of their dendrite arbors including dendrite branch pattern and orientation over multiple cycles of dendrite degeneration and regeneration (Figure 1B). Purkinje cells initially develop multiple dendritic protrusions from the soma, designated as perisomatic dendrites, before birth (Sotelo and Dusart, 2009; Takeo et al., 2015). Next, over 2–3 days Purkinje cells eliminate all perisomatic dendrites. Finally, they regenerate single stem dendritic branches over several weeks. In the course of single stem dendrite development, Purkinje cells initially develop multiplanar dendrites that extend dendritic arbors into a 3D space (Kaneko et al., 2011). However, within the next several days, Purkinje dendritic branches become trimmed and are eventually confined into a single plane (Figure 1B).

During early postnatal development, certain types of pyramidal neurons in the mammalian cortex likewise exhibit highly dynamic rearrangement of dendritic arbors. For example, layer IV pyramidal neurons in the rodent barrel cortex can be divided into two distinct subtypes: the spiny stellate neurons and the star pyramidal neurons (Nakazawa et al., 2018). The spiny stellate neurons on the edge of the barrel predominantly form synaptic contacts with thalamocortical axons (Espinosa et al., 2009; Mizuno et al., 2014; Nakazawa et al., 2018). *In vivo* two-photon imaging indicates that this organization involves selective degeneration of a subset of nascent dendrites. Spiny stellate neurons typically extend both apical and basal dendrites by postnatal day 2–3 and then retract all of their apical dendrites over the next several days while basal dendrites continue to extend, eventually forming synaptic contacts with the thalamocortical axons (Nakazawa et al., 2018; Figure 1C). Interestingly, the extension of the basal dendrites is biased to the center direction of the barrel, presumably because the thalamocortical axons mainly innervate the central part of the barrel, which recruits the basal dendrites of the spiny stellate neurons (Figure 1C).

A novel type of nerve remodeling is recently reported in the dorsal root ganglion (DRG) neurons, the nociceptors that sense pain and itch (Takahashi et al., 2019). Nociceptors innervate sensory terminals into the epidermis layer and form free-ending connections with epidermal cells in the skin. Intravital imaging in the rodent skin reveals that the nerve ends of nociceptors are highly motile structures that are continuously remodeled by extension, retraction, and pruning (Takahashi et al., 2019; Figure 1D). The pruning of the nerve ends is likely to be vital for the formation and/or maintenance of the epidermis-nerve interaction because the nerve ends overshoot to the superficial epidermal layer in the mouse model of atopic dermatitis, which enhances pain sensation in nociceptors in the atopic dermatitis model mice (Takahashi et al., 2019).

## Developmental Dendrite Remodeling in Invertebrate Neurons

*Drosophila* dendrite arborization (da) sensory neurons provide an excellent model to study the molecular and cellular basis for dendrite remodeling as class IV da (C4da) neurons undergo extensive dendrite remodeling during metamorphosis using multiple distinct cellular strategies (Williams and Truman, 2005; Emoto, 2012; Yu and Schuldiner, 2014; Kanamori et al., 2015b). Similar to Purkinje cells in the mammalian cerebellum, C4da neurons establish space-filling type dendrites during the larval stage within a 2D space between the epidermis and the musculature (Figure 1E). During metamorphosis in which flies transit from their larval to adult form within 5 days, larval C4da dendrites are completely removed from the soma by ~24 h after pupal formation (Kuo et al., 2005; Williams and Truman, 2005). After completion of dendrite pruning, C4da neurons immediately initiate dendrite regeneration and re-establish adult-specific dendritic arbors on the epidermis by eclosion (Shimono et al., 2009; Yasunaga et al., 2010, 2015; Lyons et al., 2014; Kitatani et al., 2020). Interestingly, the dendritic arbors of adult C4da neurons are rapidly reshaped from a radial shape to a lattice-like shape within 24 h after eclosion (Yasunaga et al., 2010, 2015). This radial-to-lattice reshaping arises from rearrangement of the existing radial branches into the lattice-like pattern, rather than extensive dendrite pruning followed by regrowth of the lattice-shaped arbors over the period (Yasunaga et al., 2010).

Another good model for dendrite remodeling in invertebrates is motoneurons of the hawkmoth moth *Manduca* *sexta* (Consoulas et al., 2000). During metamorphosis, muscles of the larval abdominal body wall are replaced with newly generated adult muscles, whereas certain larval motoneurons survive metamorphosis to serve as adult motoneurons in *Manduca* (Truman and Reiss, 1976). To reestablish functional connectivity with adult muscles, motoneurons need to remodel their dendritic fields. Similar to *Drosophila* C4da neurons, *Manduca* motoneurons initially undergo dendrite regression followed by a massive extension of adult-specific trees during pupal development (Levine and Truman, 1985; Kent and Levine, 1993).

*C. elegans* sensory neurons are an emerging model system for studying the molecular basis for developmental dendrite remodeling. IL2 sensory neurons typically elaborate simple unbranched dendrites (Figure 1F). In response to adverse environmental conditions, however, IL2 neurons undergo dendrite remodeling, shifting from a bipolar to multipolar state (Schroeder et al., 2013). Intriguingly, this process is reversible: the arborized dendrites are pruned away after worms are returned to the normal (non-stressful) environment. Even in normal development, PVD sensory neurons exhibit dynamic dendrite remodeling by auto fusion between terminal branches to establish their characteristic dendritic trees (Oren-Suissa et al., 2010). Another interesting example of developmental dendrite remodeling is seen in the GABAergic DVB neurons, which display male-specific posteriorly oriented outgrowth, which changes significantly during development and shows dramatic changes that are experience- and activity-dependent (Hart and Hobert, 2018).

## Dendrite Remodeling in Pathology, Injury, and Aging

Many types of neurons progressively reduce dynamics and stabilize their dendritic arbors as they mature (Emoto et al., 2006; Parrish et al., 2007b; Koleske, 2013). However, dendritic arbors of mature neurons can undergo dramatic regeneration under pathological conditions such as epilepsy and traumatic disorder (Murphy and Corbett, 2009). For instance, brain ischemia in mice induces dendrite remodeling in CA1 pyramidal neurons (Ruan et al., 2006). Similarly, post-traumatic stress disorder (PTSD)-like symptoms in mice is associated with the brain region-specific dendrite remodeling: the total number of dendrites is decreased in the prelimbic and increased in the infralimbic cortex (Lguensat et al., 2019).

Laser ablation of a part of dendrites in *Drosophila* C4da neurons induces robust dendrite regeneration (Song et al., 2012). Interestingly, injury-induced dendrite regeneration seems to be mechanistically distinct from developmental dendrite regeneration (Tao and Rolls, 2011) as well as initial dendrite development (Thompson-Peer et al., 2016). For example, dendritic branches from the same C4da neurons typically avoid overlapping in developmental regeneration as well as initial development, whereas dendrites fail to avoid overlapping with other branches from the same neurons in the injury-induced regeneration (Emoto et al., 2004; Yasunaga et al., 2015; Thompson-Peer et al., 2016). Similar to *Drosophila* C4da neurons, laser ablation of dendrites in *C. elegans* PVD neurons at the L4 stage evokes branch regeneration responses (Oren-Suissa et al., 2017). Unlike dendrite regeneration in other organisms, severed primary dendrites grow toward each other and eventually reconnect *via* branch fusion.

Dendritic branches often degenerate as animals age, and this dendrite degeneration seems to be accelerated in aging-related neurodegenerative diseases (Lin and Koleske, 2010; Adalbert and Coleman, 2013). Aging-associated dendrite degeneration is also observed in *Drosophila* C4da neurons and *C. elegans* PVD neurons (Shimono et al., 2009; Lezi et al., 2018). In the course of aging-associated dendrite degeneration in PVD neurons, from day 1 to day 9 of adulthood, varicosity-like structures are progressively formed along the dendritic branches (Lezi et al., 2018). Further, fragmented microtubules are often observed in aged PVD dendrites, but not young dendrites. These progressive changes in dendrite morphology are similar to characteristics of the degenerating dendrites observed in mammalian and *Drosophila* neurons (Emoto et al., 2006; Kanamori et al., 2013, 2015a; Koleske, 2013), implying that the underlying molecular mechanisms might be similar.

## Temporal Control of Dendrite Remodeling

### Neural Activity in Developmental Dendrite Remodeling

In the developing mammalian nervous system, the neural activity often drives fine-tuning of the functional neural circuits through multiple cellular mechanisms including dendrite remodeling (Wong and Ghosh, 2002). Indeed, glutamate receptor (NMDA and AMPA receptors)-mediated synaptic transmission is required for dendrite remodeling in the layer IV neurons in the rodent barrel cortex (Figure 1C; Mizuno et al., 2014; Nakazawa et al., 2018). Similarly, pharmacological manipulation of afferent activity in the postnatal mice dampens the multiplanar-to-monoplanar transition of dendritic trees in Purkinje cells (Kaneko et al., 2011; Figure 1B).

In many sensory systems, the spontaneous activity generated by the sensory organ often fine-tunes connections to produce a precise nearest-neighbor relationship from sensory to higher-order neurons. For instance, *in vivo* imaging of the neonatal mouse brain reveals a propagating wave of activity from the retina through the entire visual system in the brain (Feller et al., 1996; Ackman et al., 2012; Ackman and Crair, 2014). Similarly, the spontaneous activity generated in the developing cochlea propagates to auditory brain regions (Tritsch et al., 2007). In both cases, pharmacological or genetic inhibition of the spontaneous activity disturbs functional refinement of the sensory circuits (Triplett et al., 2009). Dendrite remodeling in mitral cells is largely unaffected in mice lacking function of the olfactory cyclic nucleotide-gated (CNG) channels that are required to evoke odor-triggered signaling in mitral cells (Lin et al., 2000) although a small delay in the remodeling was observed. Furthermore, *in vivo* imaging of dendrite remodeling in mitral cells indicates that over 50% of mitral cells complete dendrite remodeling before the animals’ birth (Togashi et al., 2020), supporting the idea that odor-evoked activity in mitral cells is dispensable for dendrite remodeling. Indeed, a recent study suggested that spontaneous activity, rather than evoked activity, in the olfactory circuits might play a role in dendrite remodeling in mitral cells (Fujimoto et al., 2019).

In contrast to the vertebrate nervous system, there is little evidence supporting the role of neural activity in developmental dendrite degeneration and regeneration in invertebrates. However, several reports suggest activity-dependent mechanisms in certain types of dendrite remodeling. For example, injury-induced dendrite regeneration requires neural activity in larval C4da neurons, although the neural activity is dispensable for initial dendrite growth during embryonic/larval stages as well as developmental dendrite remodeling during metamorphosis (Thompson-Peer et al., 2016). Unlike C4da neurons, neural activity promotes dendrite growth in developing *Drosophila* motoneurons (Vonhoff et al., 2013), but it remains to be determined whether activity might act in dendrite remodeling. Studies on *Manduca* motoneurons suggest a potential role of neural activity in dendrite remodeling during metamorphosis (Duch and Levine, 2002; Duch and Mentel, 2004).

### Transcriptional Control of Developmental Dendrite Remodeling

Multiple aspects of dendrite development including remodeling processes are often under transcriptional control. Dendrite remodeling in Purkinje cells is regulated by the thyroid hormone and its receptor Retinoic acid-related orphan receptor alpha (RORα). RORα was originally identified as the gene responsible for the ataxic mutant mouse *staggerer* (Sidman et al., 1962; Gold et al., 2007). Purkinje cells in *staggerer* mutant mice exhibit atrophic, fusiform-like dendrites lacking spiny branchlets (Landis and Sidman, 1978; Soha and Herrup, 1995). Further, overexpression of RORα in wild-type Purkinje cells accelerates dendrite regression in organotypic cultures (Boukhtouche et al., 2006). These data suggest that RORα mediates the regression of dendrites in the early phase of development. Besides, recent studies suggest that RORα is required not only for the branch regression early in dendrite development but also for dendrite growth in later developmental stages through regulating expression levels of multiple different genes (Takeo et al., 2015; Hatsukano et al., 2017).

The BTB/POZ-type transcription factor BTBD3 is required for dendrite remodeling of the layer IV pyramidal neurons in the rodent barrel cortex (Matsui et al., 2013). Since BTBD3 is translocated from the cytosol to the nucleus in response to neural activity in pyramidal neurons, BTBD3 might function downstream of neural activity in dendrite remodeling. Similar dendrite remodeling defects in layer IV pyramidal neurons are observed in neurons defective for the transcription factor Lhx2 (Wang et al., 2017). Lhx2 is required for BTBD3 expression in somatosensory neurons in response to neural activity (Wang et al., 2017). Since Lhx2 is constitutively expressed in developing somatosensory neurons, Lhx2 likely functions as a permissive factor for BTBD3 expression in response to neural activity.

Dendrite remodeling in invertebrates is likewise subject to transcriptional control, with signaling by the steroid hormone ecdysone playing a key role in timing and execution of *Drosophila* C4da sensory neuron remodeling (Kuo et al., 2005; Williams and Truman, 2005). The molting hormone ecdysone is secreted from the prothoracic gland at precisely timed developmental intervals, with each peak of ecdysone triggering a major developmental transition (Yamanaka et al., 2013). One of the largest ecdysone pulses occurs at the end of larval development and triggers the initiation of metamorphosis, during which larval structures including sensory dendrites are extensively remodeled to the form they will take in the adult (Thummel, 2001). The ecdysone hormone binds to the nuclear receptor composed of a non-covalent heterodimer of two proteins, EcR and USP, which, in turn, induces multiple target genes. Among the downstream targets, the transcription factor SOX14 mediates dendrite pruning in C4da neurons as Sox14 expression is induced during the early metamorphosis in an EcR/USP-dependent manner, and *Sox14* mutant C4da neurons show defects in dendrite pruning presumably in part through inducing the E3 ligase Cullin1 (Wong et al., 2013). Although *in vivo* targets of Cullin1 in dendrite pruning remain unclear, one potential outcome might be the reduction of Akt levels in C4da neurons, leading to suppression of dendrite growth.

The transcription factor AP-1 (Jun) has been implicated in activity-dependent dendrite growth in *Drosophila* motoneurons (Hartwig et al., 2008; Vonhoff et al., 2013). Recent reports suggest that AP-1 likely acts downstream of JNK signaling in both developmental and injury-induced dendrite degeneration (Hao et al., 2019; Zhu et al., 2019), yet its transcriptional targets in dendrite remodeling remain elusive.

### MicroRNAs Trigger Developmental and Injury-Induced Dendrite Regeneration

MicroRNAs (miRNAs) have recently emerged as key factors regulating developmental timing in the nervous system (Sun et al., 2013; Shenoy and Blelloch, 2014). Although miRNAs appear to play both positive and negative roles in axon regeneration after injury (Mahar and Cavalli, 2018), roles for miRNAs in dendrite regeneration have been elusive. A recent genetic screen in *Drosophila* C4da neurons has identified the miRNA *miR-87* as a critical regulator of dendrite regeneration (Kitatani et al., 2020). *miR-87* knockout impairs dendrite regeneration after developmentally-programmed pruning, whereas *miR-87* overexpression in C4da neurons causes precocious initiation of dendrite regeneration. Genetic analyses indicate that the transcriptional repressor Tramtrack69 (Ttk69) is a functional target for *miR-87*-mediated repression as *ttk69* expression is increased in *miR-87* knockout neurons and reducing *ttk69* expression restores dendrite regeneration in *miR-87* neurons. Given that Ttk69 prevents progenitor cell differentiation by suppressing the expression of genes required for neural fate specification (Xiong and Montell, 1993; Li et al., 1997; Kniss et al., 2013), *miR-87* might suppress Ttk69 function to reactivate the “neural differentiation” program including dendrite regrowth in C4da neurons. Interestingly, *miR-87* is required for dendrite regeneration after acute injury in the larval stage, as well as developmental dendrite regeneration (Kitatani et al., 2020). Since the *miR-87* expression is upregulated in C4da neurons upon dendrite injury and functions by suppressing *ttk69* expression, the *miR-87*-mediated *ttk69* suppression is a common intrinsic mechanism to drive developmental and injury-induced dendrite regeneration.

### Intrinsic Signaling in Dendrite Degeneration and Regeneration After Injury

In the axonal degeneration after injury, namely Wallerian degeneration, numerous signaling molecules are identified including NMNATs, SARM, MAPKs, and JNKs, and their roles in the axon degeneration seem to be conserved between invertebrates and vertebrates (Gilley and Coleman, 2014; Mahar and Cavalli, 2018). By contrast, much less is known about signaling pathways in dendrite regeneration after injury. Recent studies reported that JNK signaling is involved in both developmental and injury-induced dendrite degeneration in *Drosophila* C4da neurons (Hao et al., 2019; Zhu et al., 2019). In both cases, JNK acts through the canonical downstream effectors AP-1 (Jun) and Fos, but JNK signaling might play different roles in developmental and injury-induced dendrite degeneration (Hao et al., 2019; Zhu et al., 2019).

mTOR signaling promotes dendrite regeneration as well as axon regeneration after injury in vertebrates and invertebrates (Park et al., 2008; Duan et al., 2015; Agostinone et al., 2018; Beckers et al., 2019). Interestingly, mTOR is locally upregulated through local translocation of mRNAs at the injury sites in the axon regeneration (Terenzio et al., 2018), but how an injury could trigger the local translation remains elusive. Given that mTOR is required for both axon and dendrite regeneration, similar local translation for mTOR might work in dendrite regeneration as well.

The regenerative capacity of dendrites declines with age at least in invertebrates, which is the case in *Drosophila* C4da neurons (DeVault et al., 2018) and *C. elegans* PVD neurons (Kravtsov et al., 2017). In PVD neurons, the age-dependent dendrite regeneration is inhibited in part by the Insulin/IGF1 signaling pathway (Kravtsov et al., 2017).

## Spatial Control of Dendrite Remodeling

### Extrinsic Regulation

#### Cellular Interactions

Since most axon pruning involves the removal of axons that had already made synaptic connections, axon pruning is tightly associated with synapse elimination. Indeed, repulsive signaling molecules such as Semaphorins and Ephrins are required for a large-scale axon degeneration in developing mammalian nervous systems (Riccomagno and Kolodkin, 2015). By contrast, no obvious requirement for repulsive molecules has been reported in developmental dendrite pruning. A recent study reported that a weak but significant delay in dendrite pruning in mitral cells is observed in mice lacking Sema7A and its potential receptor PlexinC1 (Inoue et al., 2018). Given that Sema7A is upregulated by the odor-evoked activity in olfactory sensory neurons and that Sema7A and PlexinC1 are both required for synapse formation (Inoue et al., 2018), Sema7A and PlexinC1 might contribute to synapse formation and/or stabilization of between sensory neuron axons and mitral cells dendrites. It remains to be elucidated whether Sema7A and PlexinC1 could contribute to the removal of the synapse connections through repulsive signaling. Indeed, the Semaphorin signaling functions in both synapse formation and disruption in the *Drosophila* giant fiber system (Godenschwege et al., 2002) and in synapse removal in murine hippocampal neurons (Liu et al., 2005).

In the case of the nerve end pruning in rodent nociceptive sensory neurons (Takahashi et al., 2019), *in vivo* imaging reveals that the nerve-epithelial interactions likely play a role in the spatial control of pruning, as the nerve ends tend to be pruned underneath the tight junctions within the epithelial cells (Takahashi et al., 2019). It is thus likely that tight junctions might provide unknown spatial cues to the nerve ends.

#### Environmental Cues

The interactions between dendrites and the extracellular matrix (ECM) have been implicated in regulating the structural plasticity of dendrites *in vivo* (Fujioka et al., 2012). For instance, blockage of the integrin-ECM interaction in retina ganglion cells or genetic ablation of the integrin-mediated signaling in adult cortical neurons causes progressive retraction of dendritic branches (Moresco et al., 2005; Marrs et al., 2006). ECM modifications in the nervous system are typically achieved by the concerted actions of several different proteases that are secreted by neurons and glial cells in vertebrates and invertebrates (Yong, 2005; Page-McCaw et al., 2007). In particular, matrix metalloproteases (MMPs) are the likely regulators in dendrite development and pathology (Sekine-Aizawa et al., 2001; Szklarczyk et al., 2002). Indeed, the dendrite reshaping of *Drosophila* C4da neurons after eclosion is triggered through ECM modification by the epithelial-derived MMP2 (Yasunaga et al., 2010). In addition to the dendrite reshaping, *Drosophila* MMP2-mediated ECM modification is associated with the reduced capacity of dendrite regeneration with aging as inhibiting MMP-2 preserves the ability of dendrite regeneration in C4da neurons as the animal aged (DeVault et al., 2018). In the mouse cerebellum, the membrane-type 5 MMP (MT5-MMP; also named MMP-24) is highly expressed in developing dendrites of Purkinje cells (Sekine-Aizawa et al., 2001), implying a potential role of MT5-MMP in PC dendrite remodeling. Importantly, MMP expression levels are elevated after nervous system injury and in several neuronal pathologies. Furthermore, after a seizure, MMP-9 mRNA is transported to dendrites and synapses in the hippocampal DG of kainic acid-treated rats (Konopacki et al., 2007). Thus, MMP-mediated EMC modification might contribute to injury and pathology-induced dendrite remodeling as well as developmental dendrite remodeling. MMP activity is required for axon degeneration and regeneration (Andries et al., 2017).

In *C. elegans* PVD neurons, an antimicrobial peptide, namely NLP-29, secreted from the epidermis drives aging-associated dendrite degeneration (Lezi et al., 2018). NLP-29 expression is increased along with aging under the control of the innate immune signaling pathway, and the secreted NPL-29 is received by the G protein-coupled receptor NPR-12 in PVD neurons (Lezi et al., 2018). As expected from its regulation by the innate immune signaling, NLP-29 is also required for the fungal infection-associated dendrite degeneration in PVD neurons (Lezi et al., 2018).

### Intrinsic Regulators

#### Caspase Activity and Intracellular Calcium Levels

Developmental dendrite degeneration is often achieved in a compartmentalized manner (Kanamori et al., 2015b; Riccomagno and Kolodkin, 2015). How neurons can compartmentalize the degeneration activities into particular branches is an important issue to be addressed. Caspases are required for dendrite pruning as well as axon degradation, and the caspase activity is typically restricted in dendritic branches during dendrite pruning in *Drosophila* C4da neurons (Kuo et al., 2006; Williams et al., 2006) and in axonal branches in Wallerian degeneration (Cusack et al., 2013; Unsain et al., 2013). In the case of axon degeneration, caspase activity is spatially determined by the expression of the Inhibitor of apoptosis protein (IAP) in the soma and dendritic branches, which suppresses caspase activity in the soma and dendritic branches, therefore confining caspase activity in the axonal compartment (Potts et al., 2003; Cusack et al., 2013; Unsain et al., 2013). Also, proteasome activity spatially controls caspases as well as IAP through local degradation in Wallerian degeneration (Potts et al., 2003; Cusack et al., 2013; Unsain et al., 2013). Though not yet determined, dendrite pruning might also utilize similar strategies to restrict caspase activity.

Another factor that functions in dendrite pruning in a compartmentalized manner is intracellular calcium (Ca^2+^). Time-lapse imaging of pruning dendrites in *Drosophila* C4da neurons reveals low frequency (~0.01 Hz) Ca^2+^ transients in dendritic branches that are destined to be pruned (Kanamori et al., 2013). Interestingly, these compartmentalized Ca^2+^transients are observed ~3 h before dendrite severing, and completely predict the location and timing of the dendrite pruning. The voltage-gated Ca^2+^ channels (VGCCs) are responsible for generating Ca^2+^ transients, and mutant C4da neurons lacking the VGCC activity show significant defects in dendrite pruning, suggesting that the dendritic Ca^2+^ transients are predominantly composed of Ca^2+^ influx through VGCCs. Given that VGCCs are activated by depolarization of membrane potential, membrane potential might be locally changed in dendritic compartments, which in turn drives Ca^2+^ transients. Subsequent calcium signaling activates the Ca^2+^-dependent protease calpains that promote dendrite degeneration cooperatively with the activity of caspases (Kuo et al., 2006; Williams et al., 2006; Kanamori et al., 2013). Unlike caspase activity, Ca^2+^ transients are restricted in particular dendritic compartments in part by physical barriers that are formed in the proximal dendrites (Kanamori et al., 2015a). Interestingly, calpains and caspases function cooperatively in both developmental and injury-induced axon degeneration in the mouse visual system (Yang et al., 2013). It is thus likely that the Ca^2+^ transient-activated protease system functions in axon degeneration as well as dendrite degeneration in invertebrates and vertebrates. Additionally, a recent article reports that the low-frequency Ca^2+^ transients drive not only dendrite pruning but also synapse pruning in the neuromuscular junctions in *Drosophila* (Vonhoff and Keshishian, 2017).

#### Rearrangement of Cytoskeletal Structures

Microtubule (MT) organization is important for both the degeneration and regeneration of dendritic trees (Rolls et al., 2020). In developmental dendrite degeneration in *Drosophila* C4da neurons, MT breakdown is the earliest detectable event in dendrite pruning (Williams and Truman, 2005; Kanamori et al., 2015a; Herzmann et al., 2018). MT breakdown and subsequent disassembly in developmental dendrite degeneration are mediated by multiple factors including Kat-L60, Fidgetin, and Par-1 (Lee et al., 2009; Tao et al., 2016; Herzmann et al., 2017). Also, MT polarity organization in dendrites is a critical factor for efficient degeneration of dendrites. Unlike mammalian dendrites, dendritic MTs exhibit the minus-end-out polarity in *Drosophila* C4da dendrites (Stone et al., 2008). Knockdown of the genes involved in the control of the dendrite MT polarity such as *patronin* and *kinesins* causes significant defects in dendrite pruning in *Drosophila* C4da neurons (Herzmann et al., 2018; Wang et al., 2019).

A recent study in *Drosophila* C4da neurons has identified the receptor tyrosine kinase-like orphan receptor (Ror) as a critical factor for dendrite regeneration after injury (Nye et al., 2020). Subsequent studies suggest that Ror promotes TM nucleation for dendritic branch growth in cooperation with the Wnt signaling pathway (Nye et al., 2020; Weiner et al., 2020).

#### Membrane Dynamics

Recent studies in invertebrate models indicate that local membrane dynamics in dendritic branches impact dendrite remodeling. In the course of dendrite pruning in *Drosophila* C4da neurons, the first morphological alterations are observable in the proximal regions of dendrites: proximal dendrites actively form varicosities and dendritic branches progressively become thinner, which eventually compartmentalizes distal parts of the dendrites (Williams and Truman, 2005; Kirilly et al., 2009; Kanamori et al., 2015a). This compartmentalization of dendritic branches is driven by local endocytosis at proximal dendrites (Kanamori et al., 2015a). Genetic inhibition of Rab5- and Shibire/Dynamin-dependent endocytosis suppresses the dendrite thinning at proximal dendrites and also impairs initiation of Ca^2+^ transients in distal dendrites, suggesting that the local membrane dynamics at proximal dendrites spatially defines dendrite pruning. In addition to the local endocytosis at proximal dendrites, global endocytosis contributes to dendrite pruning in C4da neurons in part through endosomal degradation of the L1-type cell-adhesion molecule Neuroglian (Nrg; Zhang et al., 2014; Zong et al., 2018; Krämer et al., 2019). The Nrg degradation starts from the onset of metamorphosis, and loss-of-function *nrg* mutant neurons show precocious dendrite pruning. Thus, the removal of Nrg from the cell surface acts as a prerequisite for dendrite pruning. Indeed, genetic evidence suggests that this global endocytosis for Nrg degradation functions in dendrite pruning cooperatively with the local endocytosis for the compartmentalized Ca^2+^ transients (Kanamori et al., 2015a).

The type I membrane protein EFF-1, which was originally identified as a cell fusion-promoting factor, regulates the complexity of dendritic arbors by pruning excessive dendritic branches in *C. elegans* PVD neurons (Oren-Suissa et al., 2010). Consistently, the pruning process involves not only dendrite severing and retraction but also dendrite–dendrite auto fusion. Furthermore, EFF-1 mediates dendrite repair after injury by promoting membrane fusion between elongating dendritic branches (Oren-Suissa et al., 2017). Interestingly, AFF-1 fusogen, a paralog of EFF-1 expressed in neighboring hypodermal cells but not the neuron, also contributes to dendrite repair, possibly through extracellular vesicle-cell fusion (Oren-Suissa et al., 2017). A recent report proposed that EFF-1 regulates PVD dendrite morphology in part by patterning the cell adhesion molecule SAX-7 distribution in hypodermal cells (Zhu et al., 2017). It remains to be elucidated whether similar fusogen proteins might play a role in developmental and injury-induced dendrite remodeling in other organisms.

## Future Perspectives

In the past decade, considerable progress has been made in understanding the molecular mechanisms underlying dendrite remodeling including branch regeneration and degeneration *in vivo*, but many questions remain as to how the sequential rounds of branch degeneration and regeneration in developing dendrites are regulated by coordinated actions of the identified molecules. In particular, spatial regulation of dendrite degeneration and regeneration is still largely elusive. For instance, how the layer IV pyramidal neurons could selectively degenerate and regenerate apical and basal dendrites, respectively, is unknown (Figure 1D). It is even harder to imagine how Purkinje cells can confine 3D dendritic arbors into 2D arbors (Figure 1C). To tackle these interesting but difficult questions, developing novel optogenetic tools for local manipulation of molecular activity in dendrites should be a powerful approach. It should be also useful to develop *in vivo* imaging systems to precisely monitor multiple molecular activities in dendrites. Also, studies using *Drosophila* models have provided several molecular clues that could bridge our knowledge gaps in the spatiotemporal regulation of dendrite remodeling. First, given that microRNAs are the potential factors that drive the temporal transition from dendrite degeneration to regeneration, further identification of the downstream targets should be an efficient way for further understanding of the temporal control. Second, Ca^2+^ transients and Caspase activity can be good readouts to identify molecules involved in the spatial control of dendrite compartmentalization. The field is only at the starting point in terms of understanding how the components function together in dendrite degeneration and regeneration.

## Author Contributions

All authors made intellectual contributions to the manuscript and wrote the article. All authors contributed to the article and approved the submitted version.

## Conflict of Interest

The authors declare that the research was conducted in the absence of any commercial or financial relationships that could be construed as a potential conflict of interest.
